# A systematic review of interventions for promoting active transportation to school

**DOI:** 10.1186/1479-5868-8-10

**Published:** 2011-02-14

**Authors:** Palma Chillón, Kelly R Evenson, Amber Vaughn, Dianne S Ward

**Affiliations:** 1 Department of Physical Education and Sport, University of Granada, Spain; 2 Department of Epidemiology, University of North Carolina at Chapel Hill, NC, USA; 3 Center for Health Promotion and Disease Prevention, University of North Carolina at Chapel Hill, NC, USA; 4 Department of Nutrition, University of North Carolina at Chapel Hill, NC, USA

## Abstract

**Background:**

Active transportation to school is an important contributor to the total physical activity of children and adolescents. However, active school travel has declined over time, and interventions are needed to reverse this trend. The purpose of this paper is to review intervention studies related to active school transportation to guide future intervention research.

**Methods:**

A systematic review was conducted to identify intervention studies of active transportation to school published in the scientific literature through January 2010. Five electronic databases and a manual search were conducted. Detailed information was extracted, including a quantitative assessment comparing the effect sizes, and a qualitative assessment using an established evaluation tool.

**Results:**

We identified 14 interventions that focused on active transportation to school. These interventions mainly focused on primary school children in the United States, Australia, and the United Kingdom. Almost all the interventions used quasi-experimental designs (10/14), and most of the interventions reported a small effect size on active transportation (6/14).

**Conclusion:**

More research with higher quality study designs and measures should be conducted to further evaluate interventions and to determine the most successful strategies for increasing active transportation to school.

## Introduction

Currently, there is evidence that daily activities, including active transportation to school (defined as the use of active means, such as walking and bicycling to and from school), may have important health implications for young people. Active travel has been positively associated with higher daily levels of physical activity [1,2] and higher cardiorespiratory fitness [3,4]; but rates of active transportation to school have declined dramatically over the past 30 years [5]. Initiatives such as Safe Routes to School (SRTS), the Walking School Bus (WSB), or the Walk to School (WTS) program have been implemented to increase children's walking and bicycling to school with some success.

The earliest peer-reviewed intervention study targeting walking and bicycling to and from school was published in 2003, and since then the field has progressed in the design and development of interventions. Research in this area has grown in recent years, and literature reviews have been conducted on patterns of commuting to school and relationships with physical activity and other health outcomes [6-14]. However, a comprehensive review of methodology and outcomes of interventions is lacking. Therefore, in our systematic review of active transportation to school interventions published in the scientific literature, we extracted the key components and methodology for each study and assessed its quality and effectiveness to highlight both the approaches that were most successful and issues that should be addressed in future research.

## Methods

### Search strategy

A literature search was conducted using five electronic databases: MEDLINE (PubMed), Web of Science (SCI and SSCI), SPORT Discus, Cochrane library, and the National Transportation Library. Three categories of search terms were identified: school-age children, active transportation, and interventions. Specific terms used in the search were obtained from active transportation to school review studies [6,7], from the subject headings (MeSH list) within PubMed, and from the librarian's and researchers' expertise, and then adapted for each database (see additional File 1 for more detail). In addition to these electronic databases, reference lists in review papers [6-14] and our own archives of published documents were also reviewed. All English language publications through January 2010 were included.

### Selection and review process

Once the list of potentially relevant studies was compiled, titles and abstracts were reviewed to determine if the articles met the following four inclusion criteria: 1) focus on children and adolescents (6-18 y); 2) address active transportation to school; 3) contain an intervention; and 4) include at least one outcome or indicator of active transportation or physical activity. Any disagreements in the inclusion process were resolved through discussion among authors. Data were then extracted from the articles, including descriptive information, indicators of study quality, intervention strategies employed, and effectiveness. All the data extracted were checked by two researchers; if no agreement was reached, a third author adjudicated.

### Quality assessment

The quality assessment was conducted using a standardized evaluation framework, the Evaluation of Public Health Practice Projects (EPHPP) [15]. EPHPP assesses six methodological dimensions: selection bias, study design, confounders, blinding, data collection methods, and withdrawals and dropouts, all of which feed into the calculation of a global rating. Each dimension is rated on a three-point scale: strong, moderate, or weak. Two additional methodological dimensions provided by the tool, but not involved in the global rating, are intervention integrity and analyses. The EPHPP tool was created primarily for individual level observational and clinical studies based on populations; consequently, rating criteria for some items were modified by authors to improve the suitability of the tool for the interventions included in this review. These criteria are attached in additional File 2.

### Intervention strategy framework

The intervention design for each study was examined using a standardized intervention framework: the Active Living by Design (ALBD) Community Action Model [16]. The Community Action Model is an ecologic framework with multi-level strategies to increase physical activity and has been successfully applied in studies of active transportation to school [17]. This framework outlines five strategies: *Preparation*, time deliberately taken to plan and develop strategy for an initiative; *Promotions*, educating and encouraging targeted individuals; *Programs*, organized activities that engage individuals in physical activity; *Policies*, written and unwritten rules or standards that affect physical activity; and *Physical*, projects to create opportunities and remove barriers for physical activity. The 5 P's of the Community Action Model were abstracted from intervention strategies explicitly mentioned in the text of each study, as applying to active transportation to school.

### Effectiveness assessment

The effectiveness assessment was conducted by calculating the effect size using Cohen's d. Effect size was calculated between experimental and control groups, or between baseline and follow-up for the experimental group. The calculations were individualized for each study, using standardized mean or proportion differences [18], *t *statistics, or *P *values [19-21]. This information is detailed in additional File 3. The Cohen's d was divided into five levels: trivial (Cohen's d ≤ 0.2), small (>0.2), moderate (>0.5), large (>0.8), and very large (>1.3) [22].

## Results

### Study selection

The electronic search strategy produced 6311 papers among the five databases: 949 from PubMed, 807 from Web of Science, 243 from Cochrane Library, 2802 from SPORT Discus, and 1510 from the National Transportation Library. After discarding 380 duplicates, 5931 papers remained.

From these 5931 papers, 15 papers were identified for consideration based on a review of titles and abstracts; and an additional 10 papers were located through a manual search process. These 25 were thoroughly read, and 11 were excluded in a second selection process because they failed to meet inclusion criteria. Thus, 14 intervention studies relating to active transportation to school were selected and included in this review. Because two papers from Boarnet, et al. [23,24] reported on the same intervention study, results from these two papers were presented as one.

### Study population

Characteristics of the different interventions about active transportation to school are presented in Table 1. The 14 studies took place on three continents (America, Oceania, and Europe). Eight studies were conducted in the United States (three in California, and one each in Nebraska, Utah, New Mexico, Washington, and Michigan) [23-31], three studies in Australia [32-34], and two studies in the United Kingdom [35,36]. Most of the interventions discussed in the studies focused on urban settings.

**Table 1:** Characteristics of interventions on active transportation to and from school (N = 14)

Author and country (locality)	Sample and age (y)	Intervention study design and duration	Active transportation outcome measure	Other outcome measures	Results from transportation outcomes	Results from other outcomes
Boarnet et al. [23,24] USA (cities in California)	862 parents of children age 8-11y: 486 in experimental group and 376 in control group.	Quasi-experimental design with posttest assessment at 10 experimental/control schools. Duration: 3 years (Spring 2000-Fall 2003).	Parent-reported frequency of child walking and biking to school with the question: *"Would you say that your child now walks or bicycles to school: (1) less than before the project described above was built; (2) the same amount as before the project was built; (3) more than before the project was built".*	Parent-reported whether their children's passed the SRTS project.	72% of parents stated that children walked/biked the same before and after the SRTS construction; 18% stated less and 11% stated more.	56% of parents responded that their child passed the SRTS project along their usual route to school. There was a greater increase in walking among those who passed the SRTS project (P < 0.01) after sidewalk improvements and traffic control projects (primarily traffic signals). Children who passed completed SRTS projects were more likely to show increases in walking or bicycling to school than were children who would not pass by projects (15% vs. 4%; P < 0.01).
	
	1778 parents of children age 8-11 y.	Quasi-experimental with pre-post assessment at 10 experimental schools. Duration: 3 years (Spring 2000-Fall 2003).	Parent-reported frequency of child walking and biking to school (reported above) and on-site observations on counts of walking.	Parent-reported perceptions on safety.	Children walking increased after sidewalk improvement projects in 5 school sites (from 10% to 850%). Children walking increased after traffic signal improvement projects in 2 school sites. Children walking increased after crosswalk and crosswalk signal improvement projects in 1 school site and decreased in another.	Successful implementation in 50% of the projects: - 3 sidewalk gap closure projects showed success: observed children walking exclusively on the sidewalk increased 30%, 70% and 28% before and after SR2S construction in 3 school sites. - 2 replacement of four-way stops with traffic signals showed success: more parents after the SRTS project construction reported that it would increase safety (23% increase) and 19% more parents reported that the project was important (19% increase), compared with before SRTS project construction.

Heelan KA et al. [25] USA (Midwestern community in Nebraska)	324 children: 201 age 8.1 (1.7) y in experimental group and 123 age 8.4(1.6) y in control group.	Quasi-experimental with pre- 3/5 post (May 2005, September 2005, May 2006/winter 2005, spring 2005, fall 2005, winter 2006, spring 2006) assessments with 1 control and 2 experimental schools. Duration: 2 years (2005-2006)	Child-reported frequency of walking to and from school, using large posters placed in each classroom and children simply circled a picture of a person walking, biking or riding in a car or bus each morning and afternoon during all the week (5 post assessments).	Objective-measured physical activity (3 post). BMI based on measured height and weight and skinfold thickness (3 post).	Children at experimental and control groups used similar modes of transportation in the pretest: 27% actively commuted to school and 34.5% actively commuted home from school, at least once a week. Children at experimental group actively commuted more than children at control group at each posttest assessment (P < 0.05). 70.5% children at experimental group met the Healthy People 2010 recommendations (walking 50% of the time) compared with 24.7% of children at control group (results averaged across the 2 years).	Experimental participants obtained significantly more daily physical activity than control participants (P < 0.05). Across all schools, frequent walkers obtained 25% more physical activity (P < 0.05), gained 58% less body fat (P < 0.05), and attenuated BMI by 50% (P < 0.05) compared with passive commuters. There were no statically significant differences in changes in body composition over 2 years intervention.

Jordan et al. [26] USA (Tooele County, Utah)	578 parents and 767 children age 6-11 y.	Quasi-experimental pilot study with pre-post assessment at 2 control and 2 experimental schools. Duration: 1 year (June 2005-May 2006)	Parent-reported frequency of child walking and biking to school.	BMI z-score based on measured height and weight; parent-reported physical activity and sedentary habits of child; and child-report of their own physical activity, dietary and sedentary habits, and exercise self-efficacy.	Children at experimental schools walked or biked to school more often than control children (P < 0.001), both at pre and post-test. While children in both conditions increased the days per week they walked or biked to school between pre- and post-test, the change was only significant at control schools (P < 0.001).	Children in both the experimental and control cohorts showed an increased in BMI z-score, but only significant in controls. No significant differences between experimental and control children in any of the physical activity behaviors measured. Children in experimental cohort reported drinking significantly fewer soft drinks per day than children in control cohort at posttest.

Kong et al. [27] USA (Alburquerque, New Mexico)	22 children age 5-11 y and 9 parents/relatives age 20-59 y.	Quasi-experimental, pilot study with post and while intervention assessment at 2 experimental schools. Duration: 10 weeks (spring 2006)	Parent-reported and children-reported frequency of child walking to school (post). Monitoring of students and volunteer's attendance (while intervention).	BMI percentile based on measured height and weight (pre-post); children-reported and parent/relatives-reported satisfaction (post : retrospective); parent/relative's perceptions and suggestions in a focus group (post). Lead WSB parent's perceptions in interviews (post assessment).	Children walked 3.5 days and adults volunteers walked 4 days during the week intervention. 5 of 9 parents/relatives rated that WSB increased their children's walking "a lot", 4 rated it as increased "somewhat" and none rated it as "not at all". Children reported that they walked more during the intervention.	Children and parent/relatives expressed high enthusiasm in the WSB and reported that WSB provided a supportive and safe environment to promote physical activity and social interaction. 18 children reported that during the week intervention they were playing more active games and drinking fewer sodas, 17 reported to be eating more fruits and vegetables, 16 reported to be drinking fewer juices and 15 reported to watch less TV.

McKee R et al. [35] United Kingdom (Scotland, West Dunbartonshire)	60 children age 9 y: 31 in experimental group and 29 in control group.	Quasi-experimental with pre-post assessment at 1 control and 1 experimental schools. Duration: 10 weeks (Easter-summer break)	Child-reported method of travel, route and distance to school, and distance travelled by mode, using a computerized mapping program.	Child-reported stage of behavior change, benefits, motivations and barriers for active commuting to school, using an online computerized questionnaire.	Children at experimental school increased the walking distance to school 389% and children at control school increased 17% (post inter-group P < 0.001). Children at experimental school decreased the car distance to school 57.5% and children at control school increased 1.5% (post inter-group P < 0.001).	71% of children in the experimental school progressed to a higher behavior's stage or remained in the "action" and "maintenance" stages compared with 52% of the control school, in relation to active journey to school.

Mendoza JA et al.[28] USA (Seattle, Washington)	820 children age 5-11 y: 347 in experimental group and 293,180 in two control groups.	Quasi-experimental with pre-3 post (1-month, 6-month, 12-month) assessment at 2 control and 1 experimental schools. Duration: 1 year (March 2005 - March 2006)	Child-reported frequency of walking and being driven to school. Students were asked to raise their hands once to answer: *"How did you get to school today? a) walked with an adult, b)walked without an adult, c)biked, d)by school bus, e)by metro bus, f)by carpool and g)by car*.	Monitoring of children's weekly attendance. Parent leaders and volunteer's opinion in face-to-face interviews.	Children who walked to school in both experimental and control schools in pretest were not different (p = 0.39). Children at experimental schools walked to school more than children at control schools at 1-month (p = 0.001), 6-month (p = 0.001) and 12-month post assessment (p = 0.001).	

Merom et al. [32] Australia (New South Wales)	812 parents of children age 5-12 y, 717 schools	Observational with pre-3post (every year) assessment at 265 experimental schools. Duration: 4 years (2001-2004) -4days (1st April)-.	Parent-reported frequency of child modes of commuting to/from school and participation in WSTSD, using computer-assisted telephone interview system and asking: 1) *"did your child participate in the WSTSD on Friday 5 April?" *If yes, *"What did the child do? walked all the way to school, walked part of the way to school, wore a t-shirt or tattoo and other. *And 2) *'In a usual week, how does your child get to and from school every day of the week? walk, cycle, car or bus" *(post assessment 1, year 2002).	Monitoring of attendance in WSTSD and schools data. School-reported participation, students and parent's involvement in walking, curriculum and activities (post 1, year 2002). Parent-reported attitude towards WSTSD, barriers to walking, awareness of WSTSD media campaign and participation (post 1, year 2002).	31% more children walked to school on WSTSD than a normal Friday. WSTSD increased the prevalence of walking to school by 6.8%, at a population level. The school-reported prevalence estimate of walking to school (19%), was similar to rates reported by parents (21.8%).	Over the 4 years, 53% of all primary schools in NSW had participated at least once in WSTSD and 15% had participated for at least 3 years. The overall increase in school participation from 2001 to 2004 is 66%. Significantly more schools from urban than rural regions had participated (P < 0.05). Most schools stated they would participate in next year's event because it raises awareness of road safety (84%) and reinforces students' knowledge of safe pedestrian behavior (79%). Parent's awareness, participation and additional walking on WSTSD decreased. 73% of the parents confirmed they would like their child to participate next year and 20% said 'no' because school was too far away or work commitments.

Rowland et al. [36] United Kingdom (London boroughs of Camden and Islington)	1386 children age 7-11 y: 714 in experimental group and 672 in control group.	Experimental (randomized control trial) with pre-post assessment at 11 experimental and 10 control schools. Duration: 1 school year (1997-1998).	Parent-reported frequency of child modes of commuting to/from school; survey was offered in English, Bengali, Somali, Greek, Turkish, Chinese and Albanian.	Parent-report concerns about safety on the journey to school in relation to traffic, abduction and bullying. School travel plans implementation using interview with school's head teachers (post intervention).	Frequencies of modes of transportation to and from school were similar in both experimental and control groups at pre and post-test. In post-test, experimental schools reported 70% of children walking, 24% travelling by car and 6% cycled or used public transport; in control schools 71% walked, 23% travelled by car and 7% cycled or used public transport.	2 of 11 experimental schools and 1 of 10 control schools reported having travel plans prior to the study. One year later, 9 of 11 experimental schools and 0 of 10 control schools had a written travel plan; and all these 9 experimental schools implemented some form of Safe Routes activities, compared to 4 of the 10 control schools.

Sirard et al. [29] USA (Menlo Park, California).	11 children age 8-11 y: 5 in experimental group and 6 in control group.	Experimental (randomized controlled trial) with pre-post assessment a 1 experimental and 1 control groups in 1 school. Duration: 2 months (March - April 2005)		Objective-measured physical activity during 14 days. Parent's and children's opinions about WSB, using interview (post).	Experimental children increased their moderate to vigorous physical activity during the commute time (45 minutes before school) 14 minutes/day more than control children. No significant differences were detected for other weekday periods and no significant differences were detected between groups (all P ≥ 0.40) for physical activity.	Experimental children required 10 to 36 minutes to walk to school, which was proportional to the distance travelled 0.4-1.1 km (mean 0.8 km).

Staunton et al. [30] USA (Marin County, California)	1743 students age 6-15 y	Quasi-experimental with pre and 3 post (spring-01, fall-01, spring-02) assessments at 11 experimental schools*. Duration: 2 school years (2000-2002)	Child-reported frequency and mode of travelling to school. Students raised their hands to indicate the mode of traveling that morning during 3 consecutive days and results were averaged.	None reported.	From fall 2000 to spring 2002, walking increased 64%, biking increased 114%, carpooling increased 91%, and private car use carrying one student decreased 39%.	Not applicable.

Tenbrink et al. [31] USA (Jackson, Michigan)	Students age 6-11 y	Quasi-experimental with pre-3 post (every year) assessment at 4 experimental schools*. Duration: 4 years (2004-2007)	Frequency of students walking to school; Safe Routes survey data. Participation in WTS Day.	Participation in events and programs. Other community's issues.	The number of students walking to school increased: 5% of students walked to school in 2004; 7% in 2005, 11% in 2006 and 15% in 2007. Participation in WTS Day increased from 600 in 2003 to more than 1200 in 2008.	The WSB did not sustain growth. There was improvement in physical projects, policies, and walking and biking in the community.

Wen LM et al. [33] Australia (Sydney)	1966 students age 10-12 y and their parents (N = 1606).	Experimental (randomized controlled trial) with pre-post assessment at 12 experimental and 12 control schools. Duration: 2 years (2005-2006).	Child-reported and parent-reported frequency and mode of travel to/from school. Children answered over 5 consecutive school days the questions *"how did you get to school yesterday?" *and *"how did you get home yesterday?". *Parents answered the question: *"In a usual school week, how many mornings does your child go to school by each of the following ways?"*, and a similar question on the afternoon journey from school to home.	None reported	Students walking to/from school increased in both experimental and control schools, but it increased more in the experimental group (29% vs. 19% in control; p = 0.05). Students travelling by car to school decreased more in the experimental group (42%) than in the control group (32%) (p = 0.14).	Not applicable.

Zaccari & Dirkis [34] Australia (Sydney)	243 students age 5-12 y.	Quasi-experimental (pilot study) with pre-and 4 post assessments (week-1, week-2, week-3, week-4 during the intervention) at 1 experimental school. Duration: 4 weeks (April 2001)	Child-reported frequency and mode of travel to/from school by 2 sources: survey (pretest) and monitoring using daily travel diaries of poster size that were pinned to the classroom doors and each day children recorded the mode to/from school (during intervention).	Target group's opinion using 5 focus groups, 2 one-to-one interviews and observations.	At pretest, 47% were driven to school and 14% travelled by bus. There was a 3.4% reduction in car trips and a 3.4% increase in walking trips by week 4 of the intervention. For travel to school, the number of children being driven decreased while the number walking increased, for all ages. For travel from school, the number being driven decreased for all classes, and the number walking increased only from children from 5 to 9 y.	More than 80% of children lived within walking distance (within 1 kilometer of the school). The parents assisted the Council to identify road safety problems as footpath obstructions, speeding traffic, and poor crossing facilities.


**Abbreviations**: BMI = body mass index; NA = not applicable; SRTS = Safe Routes to School; WSB = Walking School Bus; WSTSD = Walk Safely to School Day

**Sample and Age**: The sample reported corresponds to people who answered the transportation and other outcomes. So, if parents reported mode of travel of their children, the sample will be parents. The sample size provided correspond to the transportation outcome in the pretest or if it is not applicable in the posttest, and if it is not applicable the sample size correspond to the outcome with a higher sample size in the pretest measure or if it is not applicable in the posttest. When none sample size is mentioned in the study, it has not been reported, Age is reported in range if it is provided; if it is not, the average of years will be indicate. When age is not provide in the study, an estimation regarding the school were done (i.e., elementary school includes children from 6 to 11 years and middle schools from 12 to 15 years in USA and United Kingdom schools; primary schools includes 5 to 12 years in Australia). **Intervention study design**: The number of measures (pre, post..., while intervention) corresponds to the maximum number of measure times regarding all the outcomes. **Outcome measures**: Information about assessment/s for each outcome has been indicated between parentheses only when these are different from the expressed in the intervention study design's column.

*Comparisons were done on a different number of schools over time. In Staunton et al. (2001), results included 6 schools for the first school year and 7 for the second school year, but only 2 schools participated in surveys both years; analysis restricted to these 2 schools produced results similar to those shown in the table. In Tenbrink et al., (2009), number of schools increased along the time and results included 1 school the first year, 3 schools (including the previous) the second year and 4 schools (including the previous) the third and fourth year.

All the studies focused on children (from age 5 to 12 y) and were set in elementary schools. However, one study also included adolescents from middle school to age 15 y [30]. The number of participants showed high variation across the intervention studies, from two studies that reported very small samples of 11 [29] and 22 [27] participants, to four studies that reported samples between 1300 to 2000 students [24,30,33,36].

### Quality assessment

The quality assessment of the interventions was conducted using the EPHPP tool. All studies were evaluated as weak in the global rating, and an analysis of the individual items is reported in Table 2. None of the studies included a sample representative of the school population, and only two (classified as moderate in selection bias) included a school sample somewhat likely to be representative of the population [25,28]. Three studies were rated as strong, with randomized controlled trial study designs [29,33,36]. Most of the study designs were moderately rated, with quasi-experimental designs (two group pre + post [25,26,28,35] or just one group pre + post [24,30,31,34]). Three interventions were weak [23,27,32] because they had only post measures or were conducted as observational studies. Most of the interventions did not take into account confounders; the three studies that did include confounders were rated as moderate [28,36] and strong [33]. Blinding or masking in most of the studies was assessed as moderate [23-26,28-32,36], and four studies were rated as weak [27,33-35]. Mediators were not assessed in any of the examined interventions; similarly, the studies generally failed to describe their theoretical frameworks.

**Table 2:** Description, strategies and quality assessment of interventions on active transportation to and from school.

Author and country	Intervention details		Effective-ness	**Quality assessment **^**c**^						
	**Description of Intervention**	**Intervention strategies **^**a**^	**Cohen's d **^**b**^	**Selection bias**	**Study design**	**Control for confounders**	**Blinding**	**Data collection**	**Withdrawals and dropouts**	**Global rating**

Boarnet et al. [23 ,24]. USA	California's SRTS program funds traffic improvement projects. The program focused on construction projects (environmental changes aimed at increasing traffic safety) as opposed to education or traffic law enforcement.10 SRTS projects were constructed and assessed at 10 schools: 5 sidewalk improvements (construction of new sidewalks, filling gaps in the sidewalk network, construction of a walking path and the installation of curbs and curb cuts), 3 crossing improvements (adding crosswalks, installing in-pavement crosswalk lighting and installing a pedestrian activated, "count-down" street-crossing signal that warns pedestrians of the amount of time remaining to cross) and 2 traffic control improvements (installation of a traffic signal).	Projects	a) 0.221** b) -0.087*	*	a) * b) **	a) * b) NA	**	*	a) NA b) *	*

Heelan KA et al. [25]. USA.	WSB is a walk-to-school program where children walk to school in groups along a set route (and with set stops along with way), with adults essentially serving as the bus driver for supervision. An adult leader met the neighborhood children at designated walk-stops at specified times each morning and walked the group to and back school. Eight routes were created for the 2 WSB schools. The WSB was conducted during the entire academic years and was only cancelled when temperatures were below 25 or if it was raining or snowing at the scheduled walk time.	Preparation Programs	0.216**	**	**	*	**	***	*	*

Jordan et al. [26]. USA.	The Gold Medal Schools program is a school-based program that incorporates the state core curriculum for the Centers for Disease Control and Prevention school health indicators, the Healthy People 2010 Objectives, and the Division of Adolescent and School Health's school health index. The goal of the program was to establish policy and environmental supports that give students and staff more opportunities for nutritious food choices, regular physical activity, and tobacco prevention. Schools were encouraged to promote fruits and vegetables at breakfast and lunch and to participate in physical activity programs (e.g., Walk Your Child to School Day, and the President's Challenge for physical fitness). More information available at http://health.utah.gov/hearthighway/gms/.	Preparation Promotion Programs Policy Projects	NA (low effect)	*	**	*	**	**	*	*

Kong et al. [27]. USA.	A WSB was implemented in the City of Albuquerque. The Police Department was involved and ensured the safety of the route and provided parents with more confidence. The recruitment started 3 months before the intervention with dissemination of flyers, posters, articles, classroom presentations and morning announcements; a part-time WSB coordinator was hired and a lead parent volunteer - who played a pivotal role between the research team and the local community and other parent volunteers who were enrolled - was recruited. All participants and their parents met with health care providers for a physical examination and a discussion about obesity prevention at the beginning of the WSB trial. Two training for WSB parent volunteers were held and during the second training, a local police officer approved designated routes for the WSB. During the walks, 4 health themes were emphasized: get up and play hard for at least 1 hour per day, turn off your television and watch no more than 2 hours per day, eat 5 servings of fruit or vegetable per day, and reduce soda and juice intake to no more than 4 ounces per day. Participants were encouraged to talk about personal strategies for making the health behavior changes on their walks to and from school. One health theme was introduced every 2 weeks and motivational incentives were distributed the week after the message delivery.	Preparation Promotion Programs	NA	*	*	NA	*	*	**	*

McKee R et al [35]. Scotland.	Travelling Green is a school-based active travel project. The teacher, children and their families used a set of written interactive resources of 2 types: curriculum materials (a guide for teachers to support school active travel projects within the curriculum. and across a variety of topic areas in a informative and interactive way appropriate for school children) and children and family resources (set of active travel resources designed to be used by children and families at home to engage them in the project outside the formal curriculum and the primary aim of the pack was to provide practical guidance about how to plan an active journey to school. The pack contained: a customized map of the school community with path networks linking the school, main pedestrian crossing points and familiar landmarks within the community and a distance and time chart provided information about journey times on foot; weekly goal-setting activities to help children and families get ready to walk and improve active travel behaviors and generic information about walking to school).	Preparation Promotion Programs	1.214 ****(outcome: distance)	*	**	*	*	*	***	*

Mendoza JA et al. [28]. USA.	The intervention school assigned a WSB coordinator (responsible for the program) and parent volunteers. The coordinator was hired and trained and was responsible for: establish WSB routes and recruit adult volunteers and students, implement school-wide activities, distribute materials on walking to school and pedestrian safety materials, provide walk to school materials and WSB information in the school newsletter, arrange for classroom presentations on pedestrian safety, organize "Two-Feet Tuesdays'" (a weekly walk to school day), organize walking workshops and the annual walk to school community celebration and conduct and informal evaluation. The WSB routes were chosen by Feet First, school personnel and parents. Both experimental and control schools received standard information on preferred walking routes from the Seattle Public Schools, access to a district-wide school traffic and safety committee, and assistance with school safety patrols.	Preparation Promotion Programs	0.256**	**	**	**	**	*	*	*

Merom et al. [32]. Australia.	New South Wales (NSW) WSTSD is an event repeated annually 1 day in April from 2001 to 2004, managed and coordinate with educational sectors and government agencies with direct interest in children's safety, environment and health and representatives from community. The main objectives of WSTSD were to reinforce safe pedestrian behavior, to promote the health benefits of walking, and create the habit at a very young age, to reduce car dependency and to promote the use of public transport. Paid media advertising before the event promoted WSTSD three weeks before to increase parents' awareness of the campaign messages. All primary schools in NSW were invited to participate and an invitation letter was sent to all principals. Registration was voluntary. Schools that registered were sent a school kit with suggestions for promoting involvement in WSTSD, including a sample letter to parents, suggestions for the school newsletter, a list of road safety activities the school could implement and promotional material (for example, stickers, posters and some t-shirts).	Preparation Promotion Programs	0.190*	*	*	NA	**	*	NA	*

Rowland et al. [36]. United Kingdom.	Assistance and advice from a travel coordinator who had formal teaching qualifications and road safety experience, for 16 hours. Road safety problems and their solutions were identified by meeting with teachers and governors, organizing focus groups of parents and pupils and encouraging the establishment of a school travel working group. Within the working group, specific safety concerns were discussed and advice was given on the development and implementation of a travel plan. The coordinator reviewed draft travel plans and provided advice about how to obtain necessary funding. The coordinator encouraged implementation of the plans by liaison with relevant parties within the local and health authorities.	Preparation Promotion Programs	0.209**	*	***	**	**	*	***	*

Sirard et al. [29]. USA.	A WSB, followed the safest route to school based on the location of the students' homes relative to each other and the school. Students walked at their normal pace but were encouraged to stay together as a group A wagon, pulled by the study team member, was used to transport backpacks and instruments. If a student lived more than 1.6 km from the school, the parent/guardian dropped the student off at one of the other student's homes (1.1 km from school), and he or she walked the remainder of the trip.	Preparation Promotion Programs	2.9 ***** (outcome: PA)	*	***	*	**	***	***	*

Staunton et al. [30]. USA.	The SRTS in Marin County promoted walking and biking to school. Using a multipronged approach, the program identified and created safe routes to schools and invited communitywide involvement. The program had 4 paid staff: program director, educator, traffic engineer and a private consulting firm. The program relied on parent, teacher, and community volunteers to carry out the activities. The activities were: mapping SRTS, Walk and Bike to school days, frequent rider miles contest, classroom education, WSB and bike trains, Newsletter and promotions, networking and presentations on the state and national level.	Preparation Promotion Programs Projects	0.259**	*	**	*	**	*	*	*

Tenbrink et al. [31]. USA.	Project U-Turn focused on active transportation in Jackson (Michigan). The project was an integrated approach with the Active Living by Design Community Action Model and the Michigan safe Routes to School model. Preparation regular meetings, guest speakers and events took place involving youth both as audience and as components of the leadership team. Implementation: the project began with a Safe Routes initiative in local schools and then it was expanded from the schools to other destinations as worksites, churches, parks. Promotion: schools held Walk to School Day events in conjunction with Safe Routes programs; volunteers and media attention raised community awareness of active commuting in the kids and the annual Smart Commute Day was organized. Jackson's Safe route to School initiative was presented at national conferences. Programs and promotional events: Walking School Bus, Smart Commute Day, Jackson's Safe Routes program were organized and encouraged active commuting and policies and physical projects to improve accessibility. Policy and physical projects: community support and educating decision makers on the benefits of policy and physical projects to support active transportation were made. Schools requested funding for new sidewalks and a study on the financial impact of introducing pedestrian improvements and programs to replace some bus routes, and complete streets resolutions were taken at each level (city, county and metropolitan).	Preparation Promotion Programs Policy Projects	0.321** (for 1 school with 4 measures)	*	**	NA	**	*	*	*

Wen LM et al [33]. Australia.	The intervention was developed within the framework of the Health Promoting Schools Policy. The intervention's strategies included: classroom activities (professional development days for teachers; resources to assist classroom learning; information for students, parents and teachers on preparation for secondary school; pedometer-based walking activities and resources on climate change and the comparative costs of active travel and driving a car), development of school Travel Access Guides to encourage parents to go to school and work by active travel, monthly newsletters for parents and improving environments with local councils (officers assisted in reviewing safety and walkability of the schools and their vicinities and then sought to improve any identified barriers to active and safe travel). The control group received a two-year program on healthy eating at school. The program components were additional funds for teachers to develop food related activities as part of classroom learning.	Preparation Promotion Programs	0.861****	*	***	***	*	*	*	*

Zaccari & Dirkis [34]. Australia.	Pilot WTS project with these objectives: increase the number of children walking to school, to reduce the number of short car trips and to reduce traffic congestion around the school. It is a comprehensive, whole-school approach integrating health-promoting approaches across the curriculum, the school ethos and environment and building on links between the home, school and community. Elements of intervention: 1) Mapping routes to school: classes were provided with a poster-size map and surrounding area for a class exercise and children who walked indicated the route to school; 2) Road safety audit: council audited all key travel routes to school to identify road safety improvements; 3) Banner painting: benefits of walking to school were explored through 36 banners displayed around the school.; 4) Travel diary (explained in transportation measure, table 1); 5) School travel policy: school policy improved health and safety outcomes in students and encourage parents to walk their children to school providing exercise and an opportunity to practice safe pedestrian behavior; 6) Newsletters: 9 weekly newsletters were produced in term 1 of 2001, to raise awareness of the problems generated by driving to school while promoting the benefits of walking; 7) Media: the project team successfully involved the local press in promoting the benefits of walking; and 8) General school assembly: a school assembly dedicated to walking to school was held on the 6 April 2001 to coincide with the inaugural NSW 'walk safely to school day' and the final day of the pilot program.	Preparation Promotion Programs Policy	0.071*	*	**	*	*	*	NA	*

**Abbreviations**: NA = not applicable; SRTS = Safe Routes to School; WSB = Walking School Bus; WSTSD = Walk Safely to School Day

^**a **^ALBD Community Action Model framework. Only if the strategy was mentioned in the paper, was it included.

^b ^Effect size: Cohen's d values were calculated for each study (detail information about calculations is provided in the additional file 2). Effect size was calculated between experimental vs control for changes between pre and posttest, when data were provided. Effect size was calculated between pretest and post-test for the experimental group when there was no control group. Effect size was calculated between experimental and control group for one measure (preferably post-test) if data for only one measure were provided. NA indicates that there were not enough data provided for a calculation. * = trivial; ** = small; *** = moderate; **** = large; ***** = very large

^**c **^Quality assessment tool for quantitative studies (McMaster University): Effective public health practice project (EPHPP) (detail information about criteria is provided in the additional file 3). The assessment of "control for confounders" was not applicable (NA) when the study had no a control group. The assessment of "withdrawals and dropouts" was not applicable (NA) when the study had only 1 measure (pre or post). When the assessment of a component was not indicated in the tool, the lower assessment (usually weak) was set. * = weak; ** = moderate; *** = strong.

The assessment method for collecting data on the primary outcome (active transportation to school) was self-report by children [25,28,30,34,35], parents [23,24,26,32,36], or both children and parents [27,33]. Mode and frequency of transportation to school were usually asked, but the form of the questions and the way of asking them differed for each study. Only three studies reported evidence of validity [25,29] or reliability [25,26,29] for the measurement instruments, and were rated as either moderate [26] or strong [25,29]. Regarding the withdrawals and dropout criteria, only those interventions that reported more than 60% of participants completing the study obtained a strong [29,35,36] or moderate [27] rating.

In describing intervention integrity, three studies reported the percentage of participants receiving the allocated intervention [28,32,36]. Only four studies measured the consistency of the intervention [25,28,32,36], and none of the studies reported contamination that might influence the results. The unit of intervention allocation in most of the studies was the school, except for two studies where it was the individual [27,29]. The unit of analysis was the individual in nine studies, and it was the school in five others [24,31,33-35]. In two studies [30,31], the active transportation measure did not include the same schools at multiple time points. Several studies did not account for school clustering or used inappropriate statistical methods for the study design [28,31-33,35,36].

### Intervention description

Two studies included all five strategies from the Community Action Model [26,31]. Two other studies included either the four strategies of preparation, promotion, programs, and projects [30] or those of preparation, promotion, programs, and policy [34]. Half the selected studies included the strategies of preparation, promotion, and programs [27-29,32,33,35,36]. One study included only preparation and program [25], and two others included only projects [23,24].

Interventions about active transportation to school involved three main elements: schools, parents, and communities. School involvement was the common element in all the interventions, except for Boarnet et al. [23,24] which focused on infrastructure projects in the community. A majority of the interventions (n = 8) reported the involvement of the school, parents, and the community [26-28,30,32-34,36], and two studies included only school and parental involvement [29,35]. One study included both school and community involvement [31], and one study included only school involvement [25].

The economic investment in the interventions was reported in several studies. Six interventions reported that one or more people were paid for being leaders [25], staff [30], coordinators [27,28], teachers [33], or researchers [34]; one study reported payment for the media [32], and another study reported large financial support from several sources for addressing active transportation in the overall community in addition to active travel to school [31]. Moreover, a number of interventions received special government funding (SRTS) for participating and implementing the program [37].

### Effectiveness

Almost all the studies reported an increase in the percentage of active transportation to school following the interventions; however, the degree of change varied widely (3% to 64%). Two studies did not report significant improvements in active transportation to school [26,36]. Two other studies reported improvements, but in other outcomes (increased physical activity levels [29] and longer average distances walked to school [35]).

Based on the calculated Cohen's d effect size (additional File 3), three studies produced trivial effect sizes [24,32,34], six reported a small effect [23,25,28,30,31,36], two reported a large effect [33,35], and one reported a very large effect [29]. Cohen's d was not calculated for two studies [26,27] due to insufficient data. The two studies that measured physical activity [29] and the distance walked [35] both reported strong effect sizes.

## Discussion

In this review, 14 interventions that promoted active transportation to school among children and adolescents were identified. These interventions were heterogeneous, varying in size, scope, and focus. Weaknesses in the quality of various study components were identified. The most common intervention strategies employed, based on the Community Action Model framework, were preparation, promotion, and programming. Although most interventions showed some improvement in use of active transportation to school, methods used to assess change and effect sizes varied, with only three interventions having a large or very large effect size.

The heterogeneity of the intervention studies, coupled with the overall weaknesses in the quality of the study protocols, limited our ability to draw clear conclusions about which intervention strategies might be most effective. From the cross-sectional literature, it is known that interventions must address a complex and varied array of factors that influence children's modes of travel to school, including the physical environment around the school, economic characteristics of the families, social networks of the children, and cultural norms [7]. However, the lessons learned from the literature need to be more consistently applied to intervention research, and the studies in this review highlight this lack of consistency.

Numerous studies have examined specifically how the physical environment affects active transportation. Cross-sectional studies have consistently shown that distance is the strongest predictor of active transportation to school among children, with longer distances associated with lower rates of active commuting [7,10,11]. However, few of the intervention studies account for distance in their study design or analyses. Distance could be considered as part of the inclusion criteria for intervention studies to target students living within a walkable distance to school. Only McKee [35] reported targeting the intervention towards children who lived within three miles from school and who were driven to school. Presumably, interventions using WSB [25,27-29,31,34] or SRTS [23,24,30,31] were conducted within walkable distances, but none of these studies mentioned distance to school as an inclusion criterion. Additionally, studies could take distance into account in their statistical analyses, especially if randomization has not occurred. Studies with these weaknesses could underestimate their results, since more than two or three miles from school is often considered a distance not typically walked for school [35].

While the heterogeneity of the interventions in this review does not allow for clear recommendations about the most effective strategies, they do highlight the importance of getting the right groups involved and working towards a specific goal. National and international active school transportation initiatives like WTS and SRTS both emphasize the importance of identifying the goal and getting all the necessary people or groups involved; however, these aspects are not captured in the Community Action Model framework of strategies employed. The importance of this mixed involvement was highlighted in an evaluation of the United States National WTS program [38] which found that getting more groups involved was critical to expanding the program's reach and engagement. Acquiring buy-in from schools, parents, and community members can be challenging, but may be an essential component in the effectiveness and sustainability of the intervention. The interventions with the highest effectiveness [32-34] shared two common elements: a) a strong involvement of schools through principals and teachers working actively in the intervention, and b) parents receiving specific materials and being encouraged to walk. While teachers may be concerned about crowded curricula [32,36], participating in one-day events that are unproductive or unsustainable[32], or taking on the extra responsibilities related to school travel [36], parents are often concerned about child and traffic safety [7,39]. Regarding community involvement, a global intervention based on a partnership approach among the health sector, schools, and local government might be successful, because it has been shown to provide a plan of action and shared responsibilities about active transportation in the community [34]. The quality of parent, school, and community involvement, as well as interaction among these groups, such as regular meetings between school and community groups, may be among the more influential components of active transportation to school interventions.

In addition, interventions focused on active transportation to school may be more effective than those with a broader focus. For example, the Gold Medal Schools program [26] and Project U-Turn [31] both incorporated all five P's of the Community Action Model framework. The Gold Medal Schools program had broad objectives, including to promote better nutrition, regular physical activity, and tobacco prevention, and its results showed only a low, non-significant improvement in rates of active transportation to school. In comparison, Project U-Turn focused solely on active transportation, and showed an increase in rates of active transportation to school from 5% to 15% of students over four years. Of course, this comparison must be interpreted cautiously since the methodology and purposes of the two programs were different.

Active transportation to school is a relatively new research field, and intervention studies are early in their development. While interventions included in this review were hampered by many methodological flaws, as highlighted by their "weak" ratings on the quality assessment measures, the details of the individual component ratings provide insight into ways future studies can be strengthened. Based on the quality assessment instrument, only interventions using experimental designs were rated as high for their study design; however, only three of the intervention studies in this review were so rated [29,33,36]. Additionally, the school typically should be used as the unit of randomization, based on the intervention components, as was done in the studies of McKee et al. and Rowland et al. [35,36] to avoid contamination between experimental and control participants. Another limitation of most studies was that measures used to assess active transportation to school were weak and often lacked evidence of validity or reliability. They differed with regard to the content of the question (e.g., they either assessed only walking or both walking and bicycling), collection methods used (e.g., interview, questionnaire), and time frames recalled (e.g., yesterday, last week). Similar measurement issues have arisen in the literature [10,33], thus making inter-study comparisons difficult. Future studies should make use of valid and reliable tools for assessing active transportation to school. Moreover, high quality studies should assess mediators, base their interventions on a theoretical framework, and use appropriate statistical methods for their study designs.

Half of the identified interventions reported a small level of effectiveness, meaning that there was only a slight increase in active transportation to school following the intervention. However, an intervention's quality may be related positively to the produced effect size. Both studies with higher quality [29,36] reported large or very large effect sizes. Another challenge in this review was the variety of methods used to report study effectiveness, making it difficult to compare across studies. Only six of the studies [25,26,28,29,35,40] provided p-values used for determining effectiveness of their interventions. The calculation of effect size with the Cohen's d was an important contribution of this review. Therefore, future studies are encouraged to report critical data elements so that effect sizes can be calculated.

## Limitations and strengths

This review is not without limitations. First, inter-study comparisons using effectiveness ratings must be considered with caution because different formulas were used for calculating the effect size from the data provided for each study, and only effectiveness ratings calculated with the same formula are completely comparable. A second limitation relates to inclusion criteria. Although experimental designs provide the strongest evidence, we also included non-experimental designs because of the limited number of interventions about active transportation to school. A third limitation concerns the relatively scarce information about details of the interventions, including study design, measurements, and implementation of the intervention (all of which may be due to space restrictions imposed by the journals in which they were published). Fourth, we found some gaps when assessing the quality of the interventions using the EPHPP tool, because this tool was designed primarily for individually-focused studies. Our study group adapted the tool in effort to make it most useful for this review.

To our knowledge, this is the first systematic review of interventions designed to promote active transportation to school among young people. The search strategy used was based on the recommendations of Pai et al. [41] for systematic reviews. A second strength was calculating the effectiveness and examining the quality of the interventions, a methodology that provided detailed insight for future studies. Finally, the rigorous review process for selecting the studies and extracting the data, including both the effectiveness and quality assessments, is a strength.

## Conclusion

A detailed discussion of interventions to promote active transportation to and from school among young people has been provided through this review. The main findings are:

1) Existing interventions to promote active transportation to and from school are heterogeneous, due to the size, scope, and focus of the intervention and measurements.

2) Interventions with appropriate school, parent, and community involvement and that work toward a specific goal (i.e., increasing active transportation) seemed to be more effective than interventions that were broader in focus.

3) Intervention quality was often low as measured by the EPHPP tool.

4) Interventions evidenced a small but promising effectiveness in increasing active transportation to school.

A range of methodological issues need to be considered in future interventions, including experimental study designs, valid and reliable data collection methods, and appropriate statistical analysis. Future studies should examine the effect of the parental, school, and community involvement, and address the complexity of multiple factors influencing active transportation to and from school. Long-term outcomes and sustainability of the active transportation to school interventions also should be examined.

## Competing interests

The authors declare that they have no competing interests.

## Authors' contributions

PC, KE, AV and DW helped develop the literature search strategy, retrieved and reviewed studies, extracted data elements, and edited the manuscript. In addition, PC led the literature search strategy, compiled the studies, and developed the manuscript. All authors read and approved the final manuscript.

## Supplementary Material

Additional file 1 **Electronic search for the intervention studies including: database, number of references found, and terms included**. The electronic search performed to identify the studies for this review is provided in detail. The terms used to search in the five different databases used (PubMed, Web of Science, Cochrane Library, SPORT Discus and the National Transportation Library) are included. Moreover, the number of final references found in each database was likewise mentioned. Click here for file

Additional file 2 **Adjusted criteria for the quality assessment tool for quantitative studies**. This file included all the adjusted criteria performed to adapt the original tool called "Quality assessment Tool for quantitative studies from the Effective Public Health Practice Project-EPHPP-", into the identified studies in this review, since these studies were not experimental or clinical studies. Adjusted criteria were performed for selection bias, study design, confounders, blinding, data collection methods, withdrawals and drop-outs, intervention integrity, analysis and final scoring Click here for file

Additional file 3 **A summary of the calculation of effect size using Cohen's d**. A detailed explanation of how was calculated the effect size using Cohen's d for every study identified in this review has been included. Conceptual data, numerical data and formulas are provided for every study that provided enough data to calculate effect size; moreover, studies where calculation was not possible were likewise mentioned. Click here for file
