# Supplementary material for: A systematic review of interventions for promoting active transportation to school
Source: Int J Behav Nutr Phys Act. 2011 Feb 14;8:10. doi: 10.1186/1479-5868-8-10 (PMC3050785; doi:10.1186/1479-5868-8-10)
Supplement: Additional file 1 — Electronic search for the intervention studies including: database, number of references found, and terms included. The electronic search performed to identify the studies for this review is provided in detail. The terms used to search in the five different databases used (PubMed, Web of Science, Cochrane Library, SPORT Discus and the National Transportation Library) are included. Moreover, the number of final references found in each database was likewise mentioned. [file 1479-5868-8-10-S1.DOC]

Additional file 1. **Electronic search for the intervention studies including: database, number of references found and terms included.**

The electronic search was conducted through January 31^st^ 2010. Three categories of search terms were identified: 1) age, 2) active transportation, and 3) interventions. Relevant publications that contained at least one term from each of the 3 categories in the full text, were identified. Moreover, use of the search term, *school,* was restricted to title and abstract to avoid its inclusion in the author’s affiliation. The following terms were used for each category:

Age: *adolescen* OR child OR children OR youth OR student* OR pupil OR pupils AND*

Active transportation: *bike OR bikers OR biking OR bicycl* OR cycle OR cycling OR cyclist* OR commute* OR commuting OR transportation OR travel*AND*

Intervention: *intervention* OR implement* OR evaluat* OR change OR pilot OR project OR environment* OR engineer* OR encourage* OR planning OR impact OR “walk to school” OR “safe routes to school” OR “walking schoolbus” OR “walking school bus” OR “walking school buses” AND*

School: *“school”[Title/Abstract])*

The electronic search was conducted in 5 databases:

1. PubMed: 949 papers

adolescen* OR child OR children OR youth OR student* OR pupil OR pupils AND

Bike OR bikers OR biking OR bicycl* OR cycle OR cycling OR cyclist* OR commute* OR commuting OR transportation OR travel*AND

intervention* OR implement* OR evaluat* OR change OR pilot OR project OR environment* OR engineer* OR encourage* OR planning OR impact OR “walk to school” OR “safe routes to school” OR “walking schoolbus” OR “walking school bus” OR “walking school buses” AND

“school”[Title/Abstract])

1. Web of Science: 807 papers

TS=(adolescen* OR child OR children OR youth OR student* OR pupil OR pupils) AND

TS=(bike OR bikers OR biking OR bicycl* OR cycle OR cycling OR cyclist* OR commute* OR commuting OR transportation OR travel*) AND

TS=(intervention* OR implement* OR evaluat* OR change OR pilot OR project OR environment* OR engineer* OR encourage* OR planning OR impact OR (walk SAME to SAME school) OR (safe SAME routes SAME to SAME school) OR (walking SAME schoolbus) OR (walking SAME school SAME bus) OR (walking SAME school SAME buses)) AND

TS=school

1. Cochrane Library: 243 papers

**(adolescen* OR child OR children OR youth OR student* OR pupil OR pupils)** and **(bike OR bikers OR biking OR bicycl* OR cycle OR cycling OR cyclist* OR commute* OR commuting OR transportation OR travel*)** and **(intervention* OR implement* OR evaluat* OR change OR pilot OR project OR environment* OR engineer* OR encourage* OR planning OR impact OR “walk to school” OR “safe routes to school” OR “walking schoolbus” OR “walking school bus” OR “walking school buses”)** and **school** (ALL TEXT)

1. SPORT Discus: 2802 papers

TX ( adolescen* OR child OR children OR youth OR student* OR pupil OR pupils ) and TX ( bike OR bikers OR biking OR bicycl* OR cycle OR cycling OR cyclist* OR commute* OR commuting OR transportation OR travel* ) and TX ( intervention* OR implement* OR evaluat* OR change OR pilot OR project OR environment* OR engineer* OR encourage* OR planning OR impact OR “walk to school” OR “safe routes to school” OR “walking schoolbus” OR “walking school bus” OR “walking school buses” ) and (TI school OR AB school) [Show Less](javascript:showHistoryTerm('ctl00_ctl00_MainContentArea_MainContentArea_historyControl_HistoryRepeater_ctl00_showless',false))

1. National Transportation Library: 1510 papers

(kw:adolescen* OR kw:child OR kw:children OR kw:youth OR kw:student* OR kw:pupil OR kw:pupils) AND (kw:bike OR kw:bikers OR kw:biking OR kw:bicycl* OR kw:cycle OR kw:cycling OR kw:cyclist* OR kw:commute* OR kw:commuting OR kw:transportation OR kw:travel*) AND (kw:intervention* OR kw:implement* OR kw:evaluat* OR kw:change OR kw:pilot OR kw:project OR kw:environment* OR kw:engineer* OR kw:encourage* OR kw:planning OR kw:impact OR kw:"walk school" OR kw:"safe routes school" OR kw:"walking schoolbus" OR kw:"walking school bus" OR kw:"walking school buses") AND kw:school
