# Supplementary material for: A systematic review of interventions for promoting active transportation to school
Source: Int J Behav Nutr Phys Act. 2011 Feb 14;8:10. doi: 10.1186/1479-5868-8-10 (PMC3050785; doi:10.1186/1479-5868-8-10)
Supplement: Additional file 2 — Adjusted criteria for the quality assessment tool for quantitative studies. This file included all the adjusted criteria performed to adapt the original tool called "Quality assessment Tool for quantitative studies from the Effective Public Health Practice Project-EPHPP-", into the identified studies in this review, since these studies were not experimental or clinical studies. Adjusted criteria were performed for selection bias, study design, confounders, blinding, data collection methods, withdrawals and drop-outs, intervention integrity, analysis and final scoring [file 1479-5868-8-10-S2.DOC]

Additional file 2**: Adjusted criteria for the quality assessment tool for quantitative studies.**

The quality assessment tool for quantitative studies (Quality assessment Tool for quantitative studies from the Effective Public Health Practice Project -EPHPP-) [1] was created to standardly evaluate observational and clinical studies based on study designs focused at the individual level. This tool was applied to the studies under review, some of which had a group-based design. In order to adapt the tool to the selected studies, several criteria were modified, as described below.

1. SELECTION BIAS

The first question (Q1) was adapted to the school level. The question used was: “Are the schools selected to participate in the study likely to be representative of the target population?”

The second question (Q2) was adapted to the school level using this question: “What percentage of selected schools agreed to participate?” If school level data was not reported or if the study had only individual level data, we reported on the individuals that reported the main outcome (active transportation to school). If both parents and children reported the main outcome, then parent’s data were used.

1. STUDY DESIGN

The design of a group randomized controlled trial was included in the first category, in the same level as randomized controlled trial studies.

The third category was modified from “cohort analytic (two group pre + post)” to a “two group pre + post design”.

The fifth category was modified from “cohort (one group pre + post (before and after))” to a “one group pre + post (before and after) design”.

1. CONFOUNDERS

If the study did not have a control group, the assessment was not applicable (NA).

1. BLINDING

If the questionnaire was self-administered, the answer to Q1 was 3 (can’t tell).

1. DATA COLLECTION METHODS

If there was no reference to the data collection tools used in the study or no indication about validity or reliability, then it was assumed to not be available and the answer to Q1 and Q2 were 2 (no).

1. WITHDRAWALS AND DROP-OUTS

If the study had only 1 measure (pre or post), the assessment was not applicable (NA).

1. INTERVENTION INTEGRITY

If there was no indication about consistency in the study, it was assumed to not be reported or available and the answer to Q2 was 2 (no).

If there was no indication about whether the participants received an unintended intervention that may influence the results, then it was assumed to not be available and the answer to Q3 was 2 (no).

1. ANALYSES

The unit of analysis in Q2 was organization/institution when results were organized for each school, and it was individual when results were organized without separating schools.

When statistical analyses were not provided, Q3 was answered by reviewing the results for each study.

1. FINAL SCORING When the final assessment of a component was not indicated in the dictionary as a possible answer, it was compared to the closest answers and the lower assessment (usually weak) was set, adopting a conservative decision.

**References**
