# Supplementary material for: A systematic review of interventions for promoting active transportation to school
Source: Int J Behav Nutr Phys Act. 2011 Feb 14;8:10. doi: 10.1186/1479-5868-8-10 (PMC3050785; doi:10.1186/1479-5868-8-10)
Supplement: Additional file 3 — A summary of the calculation of effect size using Cohen's d. A detailed explanation of how was calculated the effect size using Cohen's d for every study identified in this review has been included. Conceptual data, numerical data and formulas are provided for every study that provided enough data to calculate effect size; moreover, studies where calculation was not possible were likewise mentioned. [file 1479-5868-8-10-S3.DOC]

Additional file 3. **A summary of the calculation of effect size using Cohen´s d**

The effect size using Cohen’s d values were calculated for each study when enough data were provided. Manual calculations (for studies with no control group) and a meta-calculator were used (<http://www.lyonsmorris.com/ma1/index.cfm>) based on calculations from Cohen [1] and Abramowitz and Stegun [2].

The effect size was calculated between experimental and control groups for changes between baseline and follow-up, when data were provided. If differences between baseline and follow-up were not provided, the effect size was calculated between experimental and control groups for the posttest only. If there was not control group in the study, the effect size was calculated between baseline and follow-up for the experimental group.

There are different ways of calculating the effect size using Cohen’s d values provided.

1. If the mean or proportion, standard deviations, and sample sizes were provided, the calculation used the standard formula (mean/proportion differences divided by the pooled standard deviation) [1]. Effect sizes for 4 studies were calculated in 2 ways using means (McKee et al. and Sirard et al.) and proportions (Rowland et al. and Wen et al.).
2. If only proportions and sample sizes were provided, but standard deviations was not provided, then the effect size was calculated using proportions at baseline (√pq; q=1-p) and the standard formula (proportion differences divided the standard deviation) was applied. Effect size for 5 studies were calculated in this way (Boarnet et al.b, Merom et al., Staunton et al., Tenbrink et al. and Zaccari & Dirkis).
3. If t statistics or P values and sample sizes were provided, an intermediate calculation of r was performed using Cohen [1] and Abramowithz and Stegun [2] formulas. Effect sizes for 1 study used the t statistics (Boarnet et al.) and for 2 studies used P values (Heelan et al. and Mendoza et al.). In one case (Heelan et al.) the exact P value was not provide, so we assumed the most conservative significance (P=0.05).

The details on the calculation of effect size for each study are provided below in the table.

| **First author (year)** | **Calculation of Cohen´s d** | | |
| --- | --- | --- | --- |
|  | **Conceptual data** | **Numerical data** | **Formula** |
| Boarnet et al. (2005a) | Differences between those who passed completed the “Safe Route to school” project (experimental group) and those who did not pass by projects (control group), for the percentage of parents reporting that children walked or bicycled to school more after project construction. | t statistic: 5.71; N for experimental group: 486; N for control group: 376 | d = 2t / √df  (t: t statistic; df: degree of freedom) |
| Boarnet et al.(2005b) | Differences in the experimental group for the change in walking to school between pretest and postest for each school. A final weighted average Cohen´s d was calculated with the sample size from each school in the postest. These data were collected from the original report “Safe routes to School, volume 2” (Boarnet, Anderson, Day, McMillan and Alfonzo, 2003). | Xpo – Xpr (change in the proportion between posttest and pretest for each school); p (proportion) in pretest: a value for each school. | d = Xpo – Xpr / SD (for each school)  SD = √pq; q=1-p  (Xpo: percentage in postest; Xpr: percentage in pretest; SD: standard deviation; p: proportion of successes in pretest; q: proportion of failures in pretest) |
| Heelan et al. (2009) | Differences between experimental and control group for percentage of children who actively commuted to school in the postest. Pretest was similar in both experimental and control groups. | P: 0.05; N total: 324 | P 1-tailed = P / 2. Look up the associated Z in a normal probability table.  (Meta-calculator: <http://www.lyonsmorris.com/ma1/index.cfm>) |
| Jordan et al. (2009) | Calculation not applicable because the sample size for the experimental and control group was not provided. Because P values were provided and the improvement is higher in the control students compared to the experimental students, we could assume that the effect size would be very low and in the opposite direction. |  |  |
| Kong et al. (2009) | Calculation not applicable because quantitative values of the prevalence of active walking to school were not provided. |  |  |
| McKee et al. (2007) | Differences between experimental and control group for the change in the distance by walking between pre and posttest. | Xe (SD) for experimental group: 620 (586); Xc (SD) for control group: 47 (242); N for experimental group: 29; N for control group: 26. | d = Xe – Xc / SDp  SDp = (Ne * SDe) + (Nc * SDc) / N total  (Xe: mean in experimental group; Xc: mean in control group; SDp: standard deviation pooled; Ne: sample in experimental group; SDe: standard deviation in experimental group; Nc: sample in control group; SDc: standard deviation in control group; N: sample size) |
| Mendoza et al. (2009) | Differences between experimental and control group for the percentage of children walking to school in the posttest. Pretest was similar in both experimental and control groups. | P: 0.001; N total: 643 | P 1-tailed = P / 2. Look up the associated Z in a normal probability table.  (Meta-calculator: [*http://www.lyonsmorris.com/ma1/index.cfm*](http://www.lyonsmorris.com/ma1/index.cfm)*)* |
| Merom et al. (2005) | Differences in the experimental group (there was no control group) for the change in proportion in walking between the Walk to School Day (Xwtsd) and a usual day (Xuday). | Xwtsd – Xuday (change in the proportion): 6.8%; p (proportion) in pretest: 15% | d = Xwtsd – Xuday / SD  SD = √pq; q=1-p  (Xwtsd: percentage in the WTSD; Xuday: percentage in normal day; SD: standard deviation; p: proportion of successes in pretest; q: proportion of failures in pretest) |
| Rowland et al. (2003) | Differences between experimental and control group for the change in the percentage of children walking to school between pre and posttest. | Xe (SD) for experimental group in pretest: 65 (22); and Xe in postest :70; Xc (SD) for control group in pretest: 70(16) and Xc in postest :71; Ne for experimental group: 714; Nc for control group: 612 | d = Xe – Xc / SDp  SDp = (Ne * SDe) + (Nc * SDc) / N total  (Xe: percentage of change in experimental group; Xc: percentage of change in control group; SDp: standard deviation pooled; Ne: sample in experimental group; SDe: standard deviation in experimental group; Nc: sample in control group; SDc: standard deviation in control group) |
| Sirard et al. (2008) | Differences between experimental and control group for the change in physical activity levels increased between pretest and posttest. | Xe (SD) for experimental group: 30(10); Xc (SD) for control group: 1 (10); N for experimental group: 5; N for control group: 6 | d = Xe – Xc / SDp  SDp = (Ne * SDe) + (Nc * SDc) / N total  (Xe: mean in experimental group; Xc: mean in control group; SDp: standard deviation pooled; Ne: sample in experimental group; SDe: standard deviation in experimental group; Nc: sample in control group; SDc: standard deviation in control group) |
| Staunton et al. (2003) | Differences in the experimental group for the change (in proportion) in walking to school between pretest and posttest. | Xpo – Xpr (change in the proportion): 9%; p (proportion) in pretest: 14% | d = Xpo – Xpr / SD  SD = √pq; q=1-p  (Xpo: percentage in posttest; Xpr: percentage in pretest; SD: standard deviation; p: proportion of successes in pretest; q: proportion of failures in pretest) |
| Tenbrink et al. (2009) | Differences in the experimental group for the change (in proportion) in walking to school between pretest and posttest. Because only 1 school had data from the first measure (2004) through the last measure (2005, 2006 and 2007), so only this school´s data was used. | Xpo – Xpr (change in the proportion): 7%; p (proportion) in pretest: 5% | d = Xpo – Xpr / SD  SD = √pq; q=1-p  (Xpo: percentage in posttest; Xpr: percentage in pretest; SD: standard deviation; p: proportion of successes in pretest; q: proportion of failures in pretest). |
| Wen et al. (2008) | Differences between the experimental and control group for change in percentage of students walking to school between pretest and posttest. | Xe (SD) for experimental group : 28.8 (13.8); Xc (SD) for control group: 19.0 (8.3); N for experimental group: 403; N for control group: 404 | d = Xe – Xc / SDp  SDp = (Ne * SDe) + (Nc * SDc) / N total  (Xe: percentage in experimental group; Xc: percentage in control group; SDp: standard deviation pooled; Ne: sample in experimental group; SDe: standard deviation in experimental group; Nc: sample in control group; SDc: standard deviation in control group) |
| Zaccari & Dirkis (2003) | Differences in the experimental group (there is no control group) for the change (in proportion) in walking to school between pretest and posttest. | Xpo – Xpr (change in the proportion): 3.4%; p (proportion) in pretest: 35.4% | d = Xpo – Xpr / SD  SD = √pq; q=1-p  (Xpo: percentage in postest; Xpr: percentage in pretest; SD: standard deviation; p: proportion in pretest; q: proportion of failures in pretest). |

**References**

1. Cohen J: **Statistical power analysis for the behavioral sciences, Hillsdale, NJ: L.** In *Book Statistical power analysis for the behavioral sciences, Hillsdale, NJ: L* (Editor ed.^eds.). City: Erlbaum Associates; 1988.

2. Abramowitz M, Stegun I: *Handbook of mathematical functions with formulas, graphs, and mathematical tables.* Dover publications; 1964.
